# Clinical Characteristics and Outcomes of Decompensated Cirrhosis Patients Admitted to Hospitals With Acute Pulmonary Embolisms: A Nationwide Analysis

**DOI:** 10.7759/cureus.24162

**Published:** 2022-04-15

**Authors:** Mohammad Darweesh, Mahmoud M Mansour, Metri Haddaden, Rami Dalbah, Ratib Mahfouz, Hisham Laswi, Adham E Obeidat

**Affiliations:** 1 Internal Medicine, East Tennessee State University, Johnson City, USA; 2 Internal Medicine, University of Missouri School of Medicine, Columbia, USA; 3 Internal Medicine, MedStar Union Memorial Hospital, Baltimore, USA; 4 Internal Medicine, Kent Hospital/Brown University, Warwick, USA; 5 Internal Medicine, John H. Stroger, Jr. Hospital of Cook County, Chicago, USA; 6 Internal Medicine, University of Hawaii, Honolulu, USA

**Keywords:** live cirrhosis, acute pulmonary embolism, pulmonary embolism (pe), decompensated cirrhosis, decompensated liver cirrhosis

## Abstract

Introduction: Cirrhosis is a significant cause of mortality and morbidity worldwide. Recent studies suggested that cirrhosis is associated with an increased risk of venous thromboembolism (VTE), which disproves the old belief that chronic liver disease coagulopathy is considered protective against VTE. We conducted a retrospective study which is to our knowledge the first of its kind to assess clinical characteristics and outcomes of decompensated cirrhosis (DC) patients admitted with acute pulmonary embolism (APE).

Methodology: We used the National Inpatient Sample database for the years 2016-2019. All adults admitted to the hospitals with a primary diagnosis of APE were included. Patients less than 18 years old, missing race, gender, or age were excluded. Patients were divided into two groups, either having DC or not. A multivariate logistic regression model was built by using only variables associated with the outcome of interest on univariable regression analysis at P < 0.05.

Results: 142 million discharges were included in the NIS database between the years 2016 and 2019, of which 1,294,039 met the study inclusion criteria, 6,200 patients (0.5%) had DC. For adult patients admitted to the hospitals with APE, odds of inpatient all-cause mortality were higher in the DC group than in patients without DC; OR of 1.996 (95% CI, 1.691-2.356, P-value < 0.000). Also, vasopressor use, mechanical ventilation, and cardiac arrest were more likely to occur in the DC group, OR of 1.506 (95% CI, 1.254-1.809, P-value < 0.000), OR of 1.479 (95% CI, 1.026-2.132, P-value 0.036), OR of 1.362 (95% CI, 1.050-1.767, P-value 0.020), respectively. In addition, DC patients tend to have higher total hospital charges and longer hospital length of stay, coefficient of 14521 (95% CI, 6752-22289, P-value < 0.000), and a coefficient of 1.399 (95% CI, 0.848-1.950, P-value < 0.000), respectively.

Conclusion: This study demonstrates that DC is a powerful predictor of worse hospital outcomes in patients admitted with APE. An imbalance between clotting factors and natural anticoagulants produced by the liver is believed to be the primary etiology of thrombosis in patients with DC. The burden of APE can be much more catastrophic in cirrhotic than in non-cirrhotic patients; therefore, those patients require closer monitoring and more aggressive treatment.

## Introduction

Liver cirrhosis is a significant cause of mortality and morbidity, representing the 11th cause of death each year (with more than 1 million deaths per year) worldwide and the 12th leading cause of death in the US [1,2]. Cirrhosis results from different mechanisms of liver injury that lead to inflammation and progressive fibrosis, distorting the hepatic architecture and the formation of regenerative nodules. These structural changes result in increased resistance to portal venous flow and subsequently portal hypertension and hepatic synthetic dysfunction [3].

The natural history of liver cirrhosis can be divided into two phases: a compensated and a decompensated phase. Although this classification can oversimplify the clinical course, it is useful in terms of expecting the quality of life as well as determining the prognosis and median survival rates [4]. Decompensated cirrhosis (DC) lacks a consensus on definition; however, it is characterized by the development of cirrhosis-related complications, including ascites, hepatic encephalopathy, variceal bleeding, and spontaneous bacterial peritonitis, hepatocellular carcinoma, or hepatorenal syndrome. Despite the fact that the median survival in those patients is 2-4 years in comparison to more than 12-year median survival for those with compensated cirrhosis [4], the outcome can be unpredictable, as it ranges from the resolution of the acute decompensation to rapid progression of multiorgan failure and death [5].

In comparison to the old belief that the chronic liver disease coagulopathy is considered protective against venous thromboembolism (VTE), recent prospective [6] and retrospective studies [7,8] have suggested that individuals with cirrhosis might also be at increased risk of VTE. Furthermore, a meta-analysis enrolling a total of 695,012 cirrhotic patients and 1,494,660 non-cirrhotic controls provided a consistent result showing that cirrhosis is associated with an increased risk of VTE [9].

In this study, we investigated the effect of DC on patients admitted with acute pulmonary embolism (APE) in terms of mortality, mechanical ventilation requirement, vasopressor support, cardiac arrest, hospital length of stay, and total hospital charges. To our knowledge, this is the first study of its kind to assess clinical characteristics and outcomes of DC patients admitted with APE.

## Materials and methods

Data source

This retrospective study was conducted utilizing the National (Nationwide) Inpatient Sample (NIS) database for the years 2016 to 2019. NIS is the largest publicly available all-payer inpatient care database in the United States, containing data for more than seven million hospital stays. Its large sample size is ideal for developing national and regional estimates and enables analyses of rare conditions, uncommon treatments, and special populations. Data are collected from all the States participating in HCUP and represent more than 97 % of the United States Population [10]. The NIS includes clinical and non-clinical variables for each hospital stay, including up to 40 discharge diagnoses, and 25 procedures using the International Classification of Diseases, Tenth Revision, Clinical Modification/Procedure Coding System (ICD-10). Since NIS is de-identified data that is publicly available, it is exempt from IRB review and approval.

Study population

Patients with APE were identified using ICD-10 diagnosis codes I2602, I2609, I2690, I2692, I2693, I2694, and I2699 from all listed primary discharge diagnoses. Chronic PE and septic PE were excluded. Patients younger than 18 years of age, were admitted for elective reasons, and those with missing information for age, gender, or race were excluded, too. Patients with DC were also identified using ICD 10 codes K7030, K7460, K7469, or K7031, with one of those I8501, I8511, K652, R188, K767, K7681, C220, K7290, K7291, K7211, K7210, K7201, K7200, K7041, K7040, and K7031. We further divided the patient population into two groups: with and without DC.

Definitions of Variables

The NIS pre-defined variables were used to identify each patient’s age (in years), gender (male or female), race (White, African American, Hispanic, Asian/Pacific Islander, Native American, or others), insurance (Medicare, Medicaid or private). The Charlson comorbidity index (CCI) was used to assess the comorbidity burden since co-morbid conditions are known to influence outcomes of hospitalization negatively. It has been a widely used index to measure the severity of comorbidity burden from administrative databases [11]. The higher score indicates a more substantial burden of comorbidity. Vasopressor’s support was identified using ICD-10 codes 3E030XZ, 3E033XZ, 3E040XZ, 3E043XZ, 3E050XZ, 3E053XZ, 3E060XZ, and 3E063XZ. Mechanical ventilation was identified using ICD-10 codes: 5A1935Z, 5A1945Z, and 5A1955Z, and cardiac arrest was identified using the ICD-10 codes 5A2204Z or 5A12012 with one of the following I469, I468, I462 or I4901. Mortality was the primary endpoint, while mechanical ventilation, vasopressor use, cardiac arrest, total hospital charge, and hospital length of stay were secondary endpoints.

Statistical analysis

Analyses were performed using STATA, version 17.0 (StataCorp LLC, College Station, TX). NIS is based on a complex sampling design that includes stratification, clustering, and weighting. This software facilitates analysis to produce nationally representative unbiased results, variance estimates, and P-values.

Univariable logistic regression analysis was used to calculate unadjusted odds ratios (ORs) for the primary and secondary outcomes. A second logistic regression model was built by using only variables that were associated with the outcome of interest on univariable regression analysis at P < 0.05 and was also used to adjust for the potential patient- and hospital-level confounders. The Fisher exact test was then utilized to compare proportions, and the student t-test was also applied to compare continuous variables. All P-values were two sided, with 0.05 as the threshold for statistical significance.

## Results

There were 142 million discharges included in the NIS database between the years 2016 and 2019, of which 1,294,039 met the study inclusion criteria: 6,200 (0.5%) had DC, and the rest 1,287,839 (95.5%) did not have DC. Compared with patients who do not have DC, patients with DC were more likely to be male, had a longer length of stay, more total hospital charges, lower mean age, and lower median annual income. Patients with DC were less likely to be insured by Medicare and had a higher Charlson Comorbidity Index score for a total score of three or above. Although these differences were statistically significant, the absolute differences were small. Demographics and patients characteristics are shown in Table 1.

**Table 1:** Demographics and patients characteristics

variable	Decompensated Cirrhosis		P-value
	Absent	Present	
Males n(%)	620,480 (48)	3,974 (64)	<0.000
Age (mean in years)	62.78	62.40	<0.000
Length of stay (mean in days)	6.32	9.27	<0.000
Total hospital charge (mean in dollars)	72,969	108,516	<0.000
RACE n(%)			<0.000
White	914,623 (71)	4,134 (67)	
African American	233,871 (18)	889 (14)	
Hispanic	85,770 (6.6)	804 (13)	
Asian/Pacific Islander	16,613 (1)	140 (2)	
Native American	5,795 (0.4)	94 (2)	
other	31,165 (2)	135 (2)	
Primary Expected Payer n(%)			<0.000
Medicare	677,789 (53)	3,022 (49)	
Medicaid	171,025 (13)	1,416 (23)	
Private	347,458 (27)	1,271 (21)	
Others	91,436 (7)	490 (8)	
Charlson Comorbidity Index n(%)			<0.000
0	339,603 (26)	0 (0)	
1	275,468 (21)	385 (6)	
2	217,000 (17)	450 (7)	
3 or more	455,637 (35)	5,364 (87)	

Mortality

For adult patients admitted to the hospital with APE and compared to patients without DC, patients with DC have 1.996 higher odds of mortality (OR, 1.996; 95% confidence interval (CI) 1.691-2.356, P-value < 0.000). As exhibited in Table 2, for each one-year increase in age, there was a 1.7% increase in odds of mortality (OR, 1.017; 95% CI, 1.015-1.019), female gender was associated with a 1.7% decreased odds of mortality compared to males but this relationship was not statistically significant (OR, 0.983; 95% CI, 0.948-1.020, P-value of 0.359).

**Table 2:** Multivariate logistic regression analysis of mortality in decompensated cirrhosis (DC) patients admitted for acute pulmonary embolism (APE)

Variable	Adjusted Odds Ratio	P-value	[95% Confidence Interval]	
			Lower	Upper
Decompensated Cirrhosis	1.996	<0.000	1.691	2.356
Age	1.017	<0.000	1.015	1.019
Female versus male	0.983	0.359	0.948	1.020
Race (compared to white race)				
African American	1.000	0.998	0.949	1.054
Hispanic	1.081	0.045	1.002	1.167
Asian/Pacific Islander	1.655	<0.000	1.456	1.880
Native American	1.290	0.062	0.987	1.685
other	1.418	<0.000	1.277	1.575
Primary Expected Payer (compared to Medicare)				
Medicaid	1.111	0.005	1.033	1.195
Private	1.070	0.020	1.011	1.133
Others	1.585	<0.000	1.426	1.761
Charlson Comorbidity Index				
1	1.873	<0.000	1.733	2.024
2	2.431	<0.000	2.248	2.629
3 or more	4.337	<0.000	4.049	4.646

Hispanic and Asian/Pacific Islander races were more likely to die when compared to white race (OR, 1.081; 95% CI, 1.002-1.167, P-value of 0.045), (OR, 1.655; 95% CI, 1.456-1.880, P-value < 0.000), respectively. African American and Native American races were not statistically significant compared to the White race. Medicaid and private insurance patients were more likely to die when compared to Medicare (OR, 1.111; 95% CI, 1.033-1.195, P-value 0.005), (OR, 1.070; 95% CI, 1.011-1.133, P-value 0.020), respectively. As CCI score increases the odds of mortality also increase; compared to a CCI score of zero, patients with a score of one, two, and three were more likely to die (OR, 1.873; 95% CI, 1.733-2.024, P-value < 0.000), (OR, 2.431; 95% CI, 2.248-2.629, P-value < 0.000), and (OR, 4.337; 95% CI, 4.049-4.646, P-value < 0.000), respectively.

Mechanical ventilation, vasopressors support, and cardiac arrest

For adult patients admitted to the hospital with APE and compared to patients without DC, patients with DC have 50% higher odds of requiring mechanical ventilation, OR of 1.506 (95% CI, 1.254-1.809, P-value < 0.000), 48% higher odds of requiring vasopressors for hemodynamic support, OR of 1.479 (95% CI, 1.026-2.132, P-value 0.036), 36% high odds of cardiac arrest, OR of 1.362 (95% CI, 1.050-1.767, P-value 0.020) as shown in Table 3.

**Table 3:** Multivariate logistic regression analysis of different outcomes in decompensated cirrhosis (DC) patients admitted for acute pulmonary embolism (APE)

Outcome	Adjusted Odds Ratio	P-value	[95% Confidence Interval]	
			Lower	Upper
Ventilation	1.506	<0.000	1.254	1.809
Vasopressors	1.479	0.036	1.026	2.132
Cardiac arrest	1.362	0.020	1.050	1.767

Length of stay and total hospital charges

For adult patients admitted to the hospital with APE and compared to patients without DC, patients with DC have 14,521 dollars higher charges, a coefficient of 14,521 (95% CI, 6752-22289, P-value < 0.000), and 1.4 days longer hospital length of stay, coefficient of 1.399 (95% CI, 0.848-1.950, P-value < 0.000) as shown in Table 4.

**Table 4:** Multivariate linear regression analysis of different outcomes in decompensated cirrhosis (DC) patients admitted for acute pulmonary embolism (APE)

Outcome	Coefficient	P-value	[95% Confidence Interval]	
			Lower	Upper
Total hospital charges	14521	<0.000	6752	22289
Hospital length of stay	1.399	<0.000	0.848	1.950

## Discussion

This study demonstrates higher odds of mortality, cardiac arrest, vasopressors, and mechanical ventilation requirement in addition to higher total charges and longer hospital length of stay in DC patients when compared to patients without such a history. The mechanism by which liver cirrhosis might increase thrombosis [9] is not clear but is thought to be secondary to imbalances between clotting factors and natural anticoagulants produced by the liver. One of the devastating complications of this imbalance is PE which carries overall mortality of 10%-30% within the first month of diagnosis in the general population [12]. The burden of PE can be much more catastrophic in cirrhotic compared to non-cirrhotic patients [13]. This might be explained by a higher comorbidity burden [14,15] as well as increased susceptibility to bacterial infection in this population [16]. Søgaard et al. and their colleagues reported that PE carries higher mortality in cirrhotic patients compared to non-cirrhotic in their nationwide cohort study that was conducted in Denmark from 1994 to 2011 [12].

In our study, the mortality rate was in concordance with what he found when analyzing the data for patients with DC compared to those without DC who presented with APE. Cardiac arrests, irrespective of the outcome, were also higher in the DC group. Hispanics in our study were more likely to die of APE with DC compared to whites, which is surprisingly in paradox with what Atiemo et al. reported in their study which showed that Hispanic patients with cirrhosis experience a survival advantage compared to those white patients [17], which might be explained by the burden of PE on this population. In general, insurance is considered protective for patients and believed to lower the risk of mortality compared to those uninsured [18]. However, to our knowledge, no comparisons among different insurance types have been conducted. Interestingly, in our study, we found certain insurance types are associated with higher mortality compared to others which might be attributed to the fact that some insurance forms cover the sicker patients population.

The mortality rate was not the only indicator that DC patients tend to do worse. We found in our study that DC patients who presented with APE tend to require mechanical ventilation and vasopressor support more than their matched comparisons without DC. Therefore, based on the latter findings, we hypothesized that these patients would have longer hospital stays and higher total hospital charges, which was confirmed after analyzing the data. Treatment of APE in cirrhotic patients remains challenging due to the increased risk of bleeding and the absence of evidence to guide the management. Most studies examining anticoagulation in cirrhotic patients have been conducted on those with portal vein thrombosis rather than PE. This might by itself increases the risk of mortality due to either overtreatment, which results in bleeding, especially in patients with varices, or undertreatment.

The strength of this study stems from the size of the sample, which makes the results more representative of the general population; the use of NIS which is the largest publicly available inpatient database gives the option to adjust all outcomes to the most common baseline characteristics of both patients and hospitals to minimize confounding factors as much as possible, and it analyzes multiple demographics for patients with APE and DC. However, there are a few limitations such as being a retrospective study, which makes it susceptible to nonrandomization. Also, the NIS database includes an administrative database, which means that administrative codes were used to identify APE, DC, and other diagnoses, leading to possible misclassifications, undercodings, or overcodings. The misclassification will, however, be seen as an error rather than a bias since it is likely to occur equally across all arms. Errors do not change the nature of the relationship between two variables; rather, statistical significance between them becomes more difficult to establish.

## Conclusions

This study investigated DC patients admitted with APE and revealed increased odds of mortality, mechanical ventilation, vasopressors support, and cardiac arrest in the DC group. In addition, total hospital charges and hospital length of stay were also higher in the DC group. An imbalance between clotting factors and natural anticoagulants produced by the liver is believed to be the primary etiology of thrombosis in patients with DC. The burden of APE can be much more catastrophic in cirrhotic than in non-cirrhotic patients which might be explained by a higher comorbidity burden as well as increased susceptibility to bacterial infection. Therefore, those patients require closer monitoring and more aggressive treatment. Treatment of APE in cirrhotic patients remains challenging due to the increased risk of bleeding and the absence of evidence to guide the management. Nevertheless, more studies are required to establish this relationship before any definitive conclusion can be made.
